# Supplementary material for: Causal Relationship between Aging and Anorexia Nervosa: A White-Matter-Microstructure-Mediated Mendelian Randomization Analysis
Source: Biomedicines. 2024 Aug 16;12(8):1874. doi: 10.3390/biomedicines12081874 (PMC11351342; doi:10.3390/biomedicines12081874)
Supplement: Supplementary file 1 [file biomedicines-12-01874-s001.zip › Supplementary Figure/Figure S8/Leaveoneout plot of MR analysis for TL to ubm-b-1632.pdf]

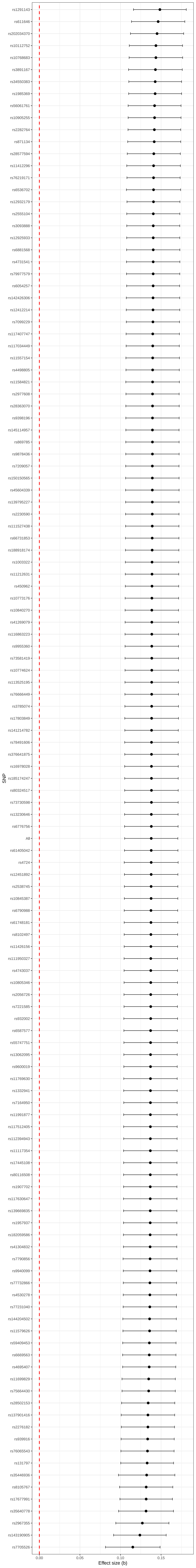

SNP
Effect size (b)
0.00
0.05
0.10
0.15
rs1291143
rs611646
rs202034370
rs10112752
rs10768683
rs3891167
rs34550383
rs1985369
rs56061761
rs10905255
rs2282764
rs871134
rs28577594
rs11412296
rs76219171
rs6536702
rs12932179
rs2555104
rs3093888
rs12925933
rs6881568
rs4731541
rs79977579
rs6054257
rs142426306
rs12412214
rs7099229
rs117407747
rs117034449
rs11557154
rs4498805
rs11584821
rs2977608
rs28363070
rs9398196
rs145114957
rs869785
rs9878436
rs7209057
rs150150565
rs45604339
rs139795227
rs2230590
rs111527438
rs66731853
rs188918174
rs1003322
rs11212631
rs450962
rs10773176
rs10840270
rs41269079
rs116863223
rs9955360
rs73581419
rs10774624
rs113525195
rs76666449
rs3785074
rs17803849
rs141214782
rs78491606
rs376641875
rs16978028
rs185174247
rs80324517
rs73730598
rs13230646
rs6776756
All
rs61405042
rs4724
rs12451892
rs2538745
rs10845387
rs6790988
rs61748181
rs8102497
rs11426156
rs111950327
rs4743037
rs10805346
rs2056726
rs7221585
rs932002
rs6587577
rs55747751
rs13062095
rs9600019
rs11769630
rs1332941
rs7164950
rs11991877
rs117512405
rs112394943
rs11117354
rs17445108
rs80116508
rs1907702
rs117630647
rs139669835
rs1957937
rs182059586
rs41304832
rs7790856
rs9940099
rs77732866
rs4530278
rs77231040
rs144204502
rs11579626
rs59409453
rs6669563
rs4695407
rs11699829
rs75664430
rs28502153
rs137901416
rs2276182
rs939916
rs76065543
rs131797
rs35446936
rs8105767
rs17677991
rs35640778
rs2967355
rs143190905
rs7705526
